# Reflecting on 20 years of breast cancer modeling in CISNET: Recommendations for future cancer systems modeling efforts

**DOI:** 10.1371/journal.pcbi.1009020

**Published:** 2021-06-17

**Authors:** Amy Trentham-Dietz, Oguzhan Alagoz, Christina Chapman, Xuelin Huang, Jinani Jayasekera, Nicolien T. van Ravesteyn, Sandra J. Lee, Clyde B. Schechter, Jennifer M. Yeh, Sylvia K. Plevritis, Jeanne S. Mandelblatt, for the Breast Working Group of the Cancer Intervention and Surveillance Modeling Network (CISNET)

**Affiliations:** 1 Department of Population Health Sciences and Carbone Cancer Center, School of Medicine and Public Health, University of Wisconsin-Madison, Madison, Wisconsin, United States of America; 2 Department of Industrial and Systems Engineering, University of Wisconsin-Madison, Madison, Wisconsin, United States of America; 3 Department of Radiation Oncology, University of Michigan Medical School, Ann Arbor, Michigan, United States of America; 4 Department of Biostatistics, University of Texas MD Anderson Cancer Center, Houston, Texas, United States of America; 5 Department of Oncology, Georgetown University Medical Center and Cancer Prevention and Control Program, Georgetown Lombardi Comprehensive Cancer Center, Washington, DC, United States of America; 6 Department of Public Health, Erasmus University Medical Center, Rotterdam, the Netherlands; 7 Department of Data Science, Dana-Farber Cancer Institute and Harvard Medical School, Boston, Massachusetts, United States of America; 8 Department of Family and Social Medicine, Albert Einstein College of Medicine, Bronx, New York, United States of America; 9 Department of Pediatrics, Boston Children’s Hospital and Harvard Medical School, Boston, Massachusetts, United States of America; 10 Department of Biomedical Data Science, Stanford University School of Medicine, Stanford, California, United States of America; Oxford, UNITED KINGDOM

## Abstract

Since 2000, the National Cancer Institute’s Cancer Intervention and Surveillance Modeling Network (CISNET) modeling teams have developed and applied microsimulation and statistical models of breast cancer. Here, we illustrate the use of collaborative breast cancer multilevel systems modeling in CISNET to demonstrate the flexibility of systems modeling to address important clinical and policy-relevant questions. Challenges and opportunities of future systems modeling are also summarized. The 6 CISNET breast cancer models embody the key features of systems modeling by incorporating numerous data sources and reflecting tumor, person, and health system factors that change over time and interact to affect the burden of breast cancer. Multidisciplinary modeling teams have explored alternative representations of breast cancer to reveal insights into breast cancer natural history, including the role of overdiagnosis and race differences in tumor characteristics. The models have been used to compare strategies for improving the balance of benefits and harms of breast cancer screening based on personal risk factors, including age, breast density, polygenic risk, and history of Down syndrome or a history of childhood cancer. The models have also provided evidence to support the delivery of care by simulating outcomes following clinical decisions about breast cancer treatment and estimating the relative impact of screening and treatment on the United States population. The insights provided by the CISNET breast cancer multilevel modeling efforts have informed policy and clinical guidelines. The 20 years of CISNET modeling experience has highlighted opportunities and challenges to expanding the impact of systems modeling. Moving forward, CISNET research will continue to use systems modeling to address cancer control issues, including modeling structural inequities affecting racial disparities in the burden of breast cancer. Future work will also leverage the lessons from team science, expand resource sharing, and foster the careers of early stage modeling scientists to ensure the sustainability of these efforts.

## Author summary

Since 2000, our research teams have used computer models of breast cancer to address important clinical and policy-relevant questions as part of the National Cancer Institute’s Cancer Intervention and Surveillance Modeling Network (CISNET). Our 6 CISNET breast cancer models embody the key features of systems modeling by incorporating numerous data sources and reflecting tumor, person, and health system factors that change over time and interact to represent the burden of breast cancer. We have used our models to investigate questions related to breast cancer biology, compare strategies to improve the balance of benefits and harms of screening mammography, and support insights into the delivery of care by modeling outcomes following clinical decisions about breast cancer treatment. Moving forward, our research will continue to use systems modeling to address issues related to reducing the burden of breast cancer including modeling structural inequities affecting racial disparities. Our future work will also leverage lessons from engaging multidisciplinary scientific teams, expand efforts to share modeling resources with other researchers, and foster the careers of early stage modeling scientists to ensure the sustainability of these efforts.

## Introduction

The purpose of systems epidemiology is to draw on expertise from different disciplines to enhance understanding of factors influencing health and disease and to use that knowledge to evaluate interventions for improving health outcomes [1–3]. Systems epidemiology uses computational tools such as simulation modeling to integrate data from numerous sources reflecting the many interdependent, multilevel influences on health and disease [3,4].

Systems modeling provides a flexible epidemiologic method to address a variety of policy and clinically relevant questions in cancer control. Since 2000, the National Cancer Institute’s Cancer Intervention and Surveillance Modeling Network (CISNET) modeling teams have developed and applied microsimulation and statistical models of several types of cancer, including breast cancer [4]. CISNET breast cancer models incorporate data on distributions of tumor characteristics, women’s risk factors, and healthcare use of breast cancer control interventions [5,6]. These models have been used to evaluate the impact of various screening and treatment interventions on multiple health outcomes in the overall United States population and population subgroups that differ by race, risk, and/or breast density [7–16].

Here, we illustrate the use of collaborative breast cancer modeling in CISNET as a systems epidemiology method. Examples are provided that demonstrate the key features of systems modeling. We also provide recommendations for future team science efforts to develop and apply systems modeling to address policy-relevant cancer prevention and control questions. This article is intended to illustrate the potential of systems modeling and highlight challenges and opportunities for systems modeling to contribute to meeting national cancer goals.

## Overview of models

### Ethics statement

No personally identifiable information was used in this study or the modeling research summarized herein. The University of Wisconsin Health Sciences Institutional Review Board determined that this study was not human subjects research (Protocol #2020–0620).

The CISNET Breast Working Group includes 6 multilevel microsimulation or analytic models: Model D (Dana Farber Cancer Institute) [17], Model E (Erasmus Medical Center) [18], Model M (MD Anderson Cancer Center) [19], Model GE (Georgetown University-Albert Einstein College of Medicine) [19], Model S (Stanford University) [20], and Model WH (University of Wisconsin-Madison and Harvard Pilgrim Healthcare Institute) [21]. All models share common inputs but were developed independently, resulting in differences in structure and underlying assumptions.

The tumor, individual person (woman), and health system levels in the models interact to provide insight into the factors affecting breast cancer incidence, mortality, and other health outcomes. The models incorporate estimates of age-specific breast cancer incidence and estrogen receptor (ER)/human epidermal growth factor receptor 2 (HER2) subtype-specific survival trends in the absence of screening or treatment and then incorporate information on how screening and molecular subtype-specific treatment patterns affect the underlying trends. Based on age-specific performance characteristics, screen detection during the preclinical screen-detectable period can result in diagnosis of earlier-stage or smaller tumors than diagnosed via symptomatic detection. Regardless of method of tumor detection, women diagnosed with breast cancer receive treatment specific to the molecular subtype of their tumors and their age at diagnosis [22].

### Model input parameters

Each modeling group begins with a common set of inputs. They may then modify the form of the inputs or use the inputs as calibration targets based on their specific model structure to best reproduce US breast cancer incidence and mortality trends as reported in the Surveillance, Epidemiology, and End Results (SEER) Program [23]. Examples of common model input parameters are described by systems level in **Table 1** with additional details available in prior publications [8,24] and online [25].

**Table 1:** Exemplar breast cancer model input parameters by level at which they are modeled in CISNET.

Parameter and level	Description	Reference
**Tumor level**		
Breast cancer incidence in the absence of screening	Estimated from age–period–cohort models for single years and ages or with a 3% annual increase from 1975 forward	[19,20,26]
Stage of breast cancer at diagnosis	SEER historical stage or AJCC stage by age group (<50, 50–64, and ≥65) by presence or absence of screening	BCSC
Distribution of ER/HER2 subtype	Molecular subtype by age (<50 and ≥50) and stage at diagnosis (AJCC or SEER Summary Stage)	BCSC
Sojourn time	Varies by decade of age and molecular subtype	[27]
Mean stage dwell time/tumor growth rate	Varies by molecular subtype, age, and stage of disease at diagnosis by model	[17,19–21]
**Person level**		
Breast density	Prevalence of breast density (BI-RADS a, b, c, d) by age group (40–49, 50–64, and ≥65)	BCSC
Risk factors	Varies by model and can include family history of breast cancer, polygenic risk, childhood cancer, and Down syndrome, among others	[9,13,15,28]
Other-cause mortality	Age at death from a cause other than breast cancer by birth cohort	[29]
Race	Race-specific incidence, mortality, screening and treatment, stage, molecular subtype, etc.	[11,12,30]
Comorbidity	Comorbidity level–specific other-cause mortality	[31]
**Health system level**		
Probability of having a mammogram	Frequency of having an annual, biennial, or irregularly spaced mammogram by decade of age and calendar year	[24]
Performance of mammography	Sensitivity of initial and subsequent mammography by age (25–39, 40–49, 50–64, and ≥65) and screening interval (annual, biennial, and irregular)	BCSC
Survival after breast cancer diagnosis in the absence of adjuvant therapy	26-year breast cancer molecular subtype-specific survival by decade of age and stage of disease or tumor size	[22]
Probability of having adjuvant breast cancer treatment	Dissemination of systemic treatment by age (<50, 50–69, and ≥70), stage at diagnosis, and molecular subtype	[24]
Hazards of reduction in mortality (or cure) with adjuvant treatment	Meta-analysis of clinical trial results by age and stage at diagnosis	[32]

AJCC, American Joint Committee on Cancer; BCSC, Breast Cancer Surveillance Consortium; BI-RADS, Breast Imaging Reporting and Data System; CISNET, Cancer Intervention and Surveillance Modeling Network; ER, estrogen receptor; HER2, human epidermal growth factor receptor 2; SEER, Surveillance, Epidemiology, and End Results.

### Tumor level

The models estimate age-specific incidence for first diagnosis of breast cancer overall and by molecular subtype over time and by birth cohort in the absence of screening [26]. Second breast cancers including recurrences and new primary breast cancers are not yet modeled but are currently being added for forthcoming studies. Five models use common estimates of breast cancer incidence rates in the absence of screening (“background incidence rate”) for each calendar year and single year of age derived from an age–period–cohort model [17,18,20,21]. One model (Model M) assumes a linear model for the annual incidence rates during the years 1975 to 2012 under a hypothetical scenario of no screening then adds the effect of screening dissemination patterns to the linear model. A Bayesian approach is applied to adjust the linear model parameters so that the model output matches the SEER rates in 1975 to 2012, resulting in an annual increase of 0.3% (SD 0.2%) of the baseline incidence in 1975 (approximately 167 per 100,000) or equivalently 0.5 cases per 100,000 women per year [19].

All 6 models consider 4 breast cancer molecular subtypes based on age-specific proportions of ER and HER2 positive and negative breast cancers in the population. Stage of breast cancer at detection with and without screening is based on data from the Breast Cancer Surveillance Consortium (BCSC) [8]. Tumors are assigned sojourn times (defined as the time from when tumors are detectable by screening until they are detectable by clinical symptoms) or mean tumor doubling times specific to subtype conditioning on age group (≤40, 40 to 50, and ≥50) [22]. Tumor growth for model WH is based on a Gompertz-type function with a lag to account for the difference in timing of detection by screening or symptoms [21]. Tumors can be detected at a younger age and earlier stage (or smaller size) if they are screen detected than if they are clinically detected.

In all models, some tumors could be considered overdiagnoses, where overdiagnosis is defined as screen-detected cancers that would not have been diagnosed within the woman’s lifetime in the absence of screening. These tumors have no effect on breast cancer–specific mortality.

### Individual person level

The models simulate the life history of each individual woman until death. At the start of the simulation, a woman is assigned a date at birth and date of death from other-cause mortality. Based on the incidence parameters described above, some women develop breast cancer and are assigned a date (and age) of symptomatic clinical detection for breast cancer in the absence of screening. If the date of breast cancer is before the date of other-cause death, the cancer can be screen or clinically detected. Women can die of non-breast cancer causes at any time; non-breast cancer mortality rates by age and calendar year are derived from national data [29].

Breast density is a radiographic feature observed on mammogram images that reflects the degree to which fibroglandular tissue is radio-lucent (white on the image) or fatty and radio-opaque (dark on the image). Women are assigned one of 4 breast density levels [Breast Imaging Reporting and Data System (BI-RADS) categories: almost entirely fat, scattered fibroglandular density, heterogeneously dense, or extremely dense] at age 40 [33]. Women are assigned to either the same breast density category or to the next lower category at ages 50 and 65 based on observed age-specific prevalence in the BCSC [34,35]. We assume density does not change after age 65. Density affects the risk of breast cancer and mammography performance [24].

### Health system level

Although women are assigned receipt of screening or therapy at the individual person level in the models, these interventions are delivered via the health system. Hence, healthcare policies at the health system level heavily influence breast cancer screening and treatment that women receive. Women are assigned an age at first mammogram and screening frequency based on the distribution observed for their birth cohort using data from the BCSC, the National Health Interview Survey, and the US Food and Drug Administration’s Mammography Quality Standards Act and Program [24]. Model inputs for mammography performance are based on data from the BCSC and depend on a woman’s age (25 to 39, 40 to 49, 50 to 64, and ≥65), density level, screening interval (annual, biennial, and triennial/infrequent), and whether the mammogram was the first screening or not [8,24].

All women diagnosed with breast cancer are assumed to receive initial therapy with mastectomy or lumpectomy with radiation, but local therapy is not explicitly modeled. Subtype-specific adjuvant treatment (chemotherapy, endocrine therapy, and trastuzumab) is assigned according to a dissemination model based on SEER patterns of care special studies (1980 to 1996) and the National Comprehensive Cancer Network Outcomes Database (1997 to 2012) [24]. Breast cancer survival depends on age group (<40, 40 to 49, 50 to 59, 60 to 69, and 70 to 84), and American Joint Committee on Cancer (AJCC)/SEER stage or tumor size in the absence of screening and treatment (background survival) as estimated from our prior research [8,22]. Systemic treatment reduces the hazards of breast cancer death (Models D, GE, M, and S) or results in cure for some cases (Models E, WH) based on age-specific data from the most current Oxford Overview of clinical trials [32]. Since the Overview did not find age differences in efficacy, hazards reductions are applied to all age groups.

### Model output analysis

The population of US women is modeled starting in the year 1975 until the most recent year of data available in the SEER Program. Either the entire population can be modeled (all ages and birth cohorts) or a single recent birth cohort can be selected to simulate expected outcomes of a contemporary cohort of women receiving the current standard of care for breast cancer screening and treatment. The models generate a wide range of benefit and harm outcomes (**Table 2**).

**Table 2:** Common outputs of the CISNET breast cancer models.

Counts	Benefits	Harms
• Proportion of women alive • Number of DCIS and invasive cancers • Stage • Number of breast cancer deaths^1^ • **Number of distant recurrences** • Number of mammograms • **Number of survivors with adverse effects** • **Surgery** • **Number of secondary cancers** • Costs	• Cancer deaths averted • % reduction in mortality • Life year gains • Quality-adjusted life year gains • **Distant recurrences avoided** • **Distant recurrence-free survival** • Breast cancer survival • **Quality-adjusted survival**	• Interval cancers • Advanced staged diagnoses^2^ • Overdiagnosis • False positives^3^ • Biopsies after false positives • **Secondary cancers** • **Adverse treatment effects**

Modeling teams are currently adding new outputs as shown in bold.

^1^Incidence and mortality rates can be age-adjusted based on the most recent US Standard Population used by SEER. Incidence is modeled over the lifetime. In future research, the models may estimate 5-year risk of developing breast cancer or risk of developing an advanced prognostic stage.

^2^Advanced stage can be defined as node positive disease (i.e., regional or stage 2b) or advanced prognostic stage.

^3^Positive mammography exam with no breast cancer diagnosed within the follow-up period.

CISNET, Cancer Intervention and Surveillance Modeling Network; DCIS, ductal carcinoma in situ; SEER, Surveillance, Epidemiology, and End Results.

Mortality reductions can be attributable to screening alone, treatment alone, or the combination in a given calendar year by calculating the difference between the mortality rates predicted with an intervention and the background mortality rate in the absence of screening and treatment. There are several methods for these estimates that consider the potential for negative synergy between the contributions of screening and systemic treatment on mortality reductions (i.e., as treatment becomes more effective at later stages, the contribution of screening to mortality reductions decreases) [8].

### Validation and uncertainty analysis

As an exercise to validate models against data that were not used in their construction and calibration, we have replicated results of the United Kingdom Age Trial of mammography screening for women in their 40s. The results demonstrated that the models closely reproduced the effect of annual mammography screening on breast cancer incidence and long-term mortality [36]. To capture model uncertainty, all analyses are reported by model and summarized as the average and range across models. The range provides a measure of structural uncertainty because each model has different assumptions and structures to represent unobservable factors such as background incidence and tumor parameters. Input parameter uncertainty is captured through inherent modeling approaches including the random selection of parameters for each simulated woman from the full range of joint parameter distributions as well as sensitivity analyses. As input parameters are updated in the models, reports featuring the new data include sensitivity analyses to demonstrate the impact on the findings after varying the new input values. While 95% credible intervals are sometimes presented around single model estimates in reports, the ranges provided by the models (when multiple models are used) provide an informative interval that describes uncertainty in modeling structure and assumptions—the dominant source of uncertainty—rather than parameter sampling error [21].

## Examples of CISNET multilevel modeling

Previous studies have demonstrated the flexibility of the CISNET breast cancer models to incorporate the complex interplay between the multiple factors on breast cancer as reflected by tumor, person, and health system levels. Here, we summarize exemplar findings by the CISNET breast cancer researchers that demonstrate these interactions.

### Tumor and health system level interactions

**Fig 1:**
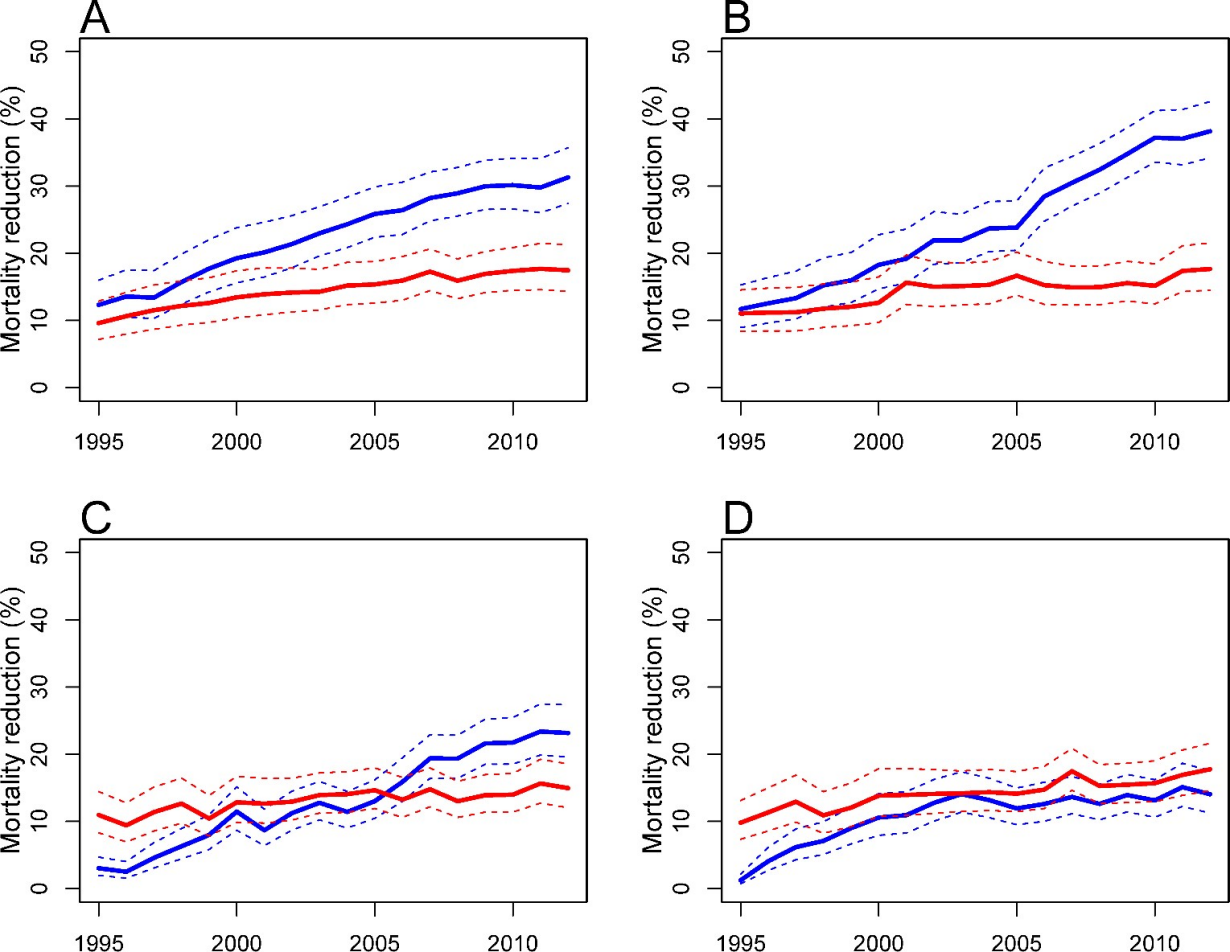
The relative reduction in breast cancer mortality (%) by treatment (blue line) and screening (red line) for the **(A)** ER+, HER2−, **(B)** ER+, HER2+, **(C)** ER−, HER2+, and **(D)** ER−, HER2− molecular subtypes with 95% credible intervals (dashed lines), 1995–2012, Model M. ER, estrogen receptor; HER2, human epidermal growth factor receptor 2. *Image source*: Plevritis et al., *JAMA* 2018 [8].

The number of treatment options for women diagnosed with breast cancer has increased dramatically over the past 2 decades. The CISNET breast cancer modelers have quantified the associations of screening and treatment with reductions in US breast cancer mortality rates. Recent work extended a previous analysis [7] by updating mortality trends considering molecular subtype and advances in systemic treatment [8]. As women in some models are diagnosed with breast cancer, their tumors are assigned a molecular subtype, while other models incorporate subtype-specific sojourn times, thus the natural history of the tumors are linked with treatments—received through the health system—and survival. Based on results from all 6 models, we found that delivery of newer adjuvant therapies resulted in greater estimated reductions in overall breast cancer mortality than screening advances from 1995 to 2012. Specifically, in 2012, the estimated reduction in overall breast cancer mortality rate was 49% (model range, 39% to 58%), with 63% (model range, 49% to 74%) of this reduction associated with treatment and 37% (model range, 26% to 51%) associated with screening. Of the 63% associated with treatment, 31% was associated with chemotherapy, 27% with endocrine therapy, and 4% with trastuzumab. The estimated relative contributions associated with screening versus treatment varied by molecular subtype (**Fig 1**). For example, for ER-negative/HER2-negative cases, the relative contributions by treatment were always lower than those by screening reflecting the lack of effective treatments for this subtype.

CISNET modeling teams have also examined linkages between the tumor and health system level interactions related to the impact of radiotherapy by simulating a clinical trial. This research conducted by Models GE and M illustrated that simulation modeling can prove useful in the modification or redesign of clinical trials. This analysis found that omission of radiotherapy in hormone-sensitive patients with low recurrence risk may lead to a modest increase in locoregional recurrence event rates, but did not appear to increase the rate of distant recurrence or death [37,38]. Model GE has also completed a series of analyses on the use of Oncotype DX to guide adjuvant chemotherapy decisions, demonstrating the power of modeling to support clinical decision-making [31,39].

As another example of the interaction between the natural history of breast cancer represented by tumor growth and the health system in the CISNET models, we have investigated several issues focused on ductal carcinoma in situ (DCIS). Following the widespread dissemination of mammography screening for breast cancer, DCIS incidence increased sharply [40]. While ongoing trials in the US and Europe are testing active surveillance [41–43], women with DCIS are generally treated in the healthcare system similar to women with local invasive breast cancer, receiving surgery and adjuvant hormonal therapy. However, the value of detecting DCIS by screening is uncertain as not all DCIS might progress to invasive breast cancer. The natural history of DCIS is largely unknown and believed to be more complex than the progression of invasive breast cancer. Published data on modeling of the DCIS natural history show large variations in model structures, assumptions, estimation methods, and data sources [44].

Two CISNET models (Models D and E) investigated the natural history of DCIS and selected 6 plausible parameterizations that could explain DCIS and invasive breast cancer incidence in the US. Using the models, they estimated mean sojourn time in the preclinical screen-detectable DCIS state, overdiagnosis of DCIS, and progression to invasive breast cancer in the absence of biopsy or complete excision [45]. Overdiagnosis of DCIS was defined as screen-detected DCIS lesion that would not have been diagnosed in the absence of screening as it would not have progressed to clinical DCIS or invasive breast cancer in the woman’s remaining lifetime. For model-specific parameters, Model D used the data from the Norwegian Breast Cancer Screening Program [46] and Model E calibrated with SEER data on DCIS [44]. Details have been reported previously [17,18,47]. Both models characterized the DCIS progression from undetectable DCIS to screen-detectable preclinical DCIS, clinical DCIS, or invasive breast cancer. Regression was allowed from the preclinical DCIS state to the “no breast cancer” state. Submodels assumed 30%, 50%, or 80% of breast lesions progress from undetectable DCIS to preclinical screen-detectable DCIS. Each model additionally allowed or prohibited DCIS regression. Estimated mean sojourn time in the preclinical screen-detectable DCIS state ranged 0.2 to 7.7 years, but mostly less than 4 years. Estimated overdiagnosis ranged 3.1% to 4.8% without regression and 13.3% to 19.3% with regression for Model D. The level of overdiagnosis was much higher in model E, ranging 35% to 66% [45].

The modeling work on DCIS shows the challenges to deciding between alternative representations of DCIS natural history and the complexity of healthcare decisions for DCIS. Main findings from Models D and E indicate that the majority of screen-detectable but unbiopsied preclinical DCIS lesions progress to invasive breast cancer and that the mean sojourn time in preclinical DCIS is relatively short. Given the heterogeneous nature of DCIS, the progression of DCIS needs to be further modeled by grade and molecular subtype to more completely reflect the key features of DCIS biology. Overall, this body of work illustrates that screening approaches for breast cancer selectively detect different tumor characteristics, and CISNET models are well suited for representing the interplay between tumor and detection features in healthcare delivery.

### Person and health system level interactions

In CISNET, we have examined several instances where person-level factors interact with health system level factors to influence breast cancer outcomes. In a first example of person and health system level interactions, we used 2 CISNET models (Models E and GE) to evaluate the benefits and harms of mammography screening based on polygenic risk (313 single nucleotide polymorphisms, SNPs [48]) and family history of breast cancer in a first-degree female relative. Models accounted for the ages at which women learned of their family member’s breast cancer diagnosis or whether women had no first-degree family history of breast cancer in their lifetimes. Screening strategies varied by initiation age (30, 35, 40, 45, and 50) and interval (annual, hybrid, biennial, and triennial). We found that women at increased risk due to a positive family history, polygenic risk, or both could benefit importantly from starting screening at an earlier age, while women at the lower end of the risk spectrum could consider screening at triennial intervals. At the population level, risk-based screening based on family history and polygenic risk was predicted to lead to a substantial increase in life years gained (154 versus 118 per 1,000 women) compared to biennial non-risk-based screening from age 50 to 74 years. We also found that risk-based screening resulted in a substantial decrease in false positives (1,169 versus 1,666) compared to American Cancer Society guideline recommended screening for all women (annual from age 45 to 54 and biennial from age 55 to 74 years) (**Table 3**). Overall, we found that using the person-level factors breast cancer family history and polygenic risk to tailor (system level) screening strategies, more breast cancer deaths can be prevented and lives extended [28]. In general, findings were consistent across the 2 models with the most variation observed for estimates of overdiagnosis.

**Table 3:** Risk-based screening strategies based on breast cancer family history, polygenic risk score, and family history combined with polygenic risk.

Screening guideline^1^	Screening strategy	Number of screens	Life years gained^2^	Breast cancer deaths averted^2^	Overdiagnoses	False positives
USPSTF	Biennial 50–74	11,182	118	6.7	14.5	920
Risk based	Family history	11,840	125	6.9	14.9	1,000
Risk based	Polygenic risk	12,990	141	7.4	16.0	1,156
Risk based	Family history and polygenic risk	13,089	154	7.9	16.6	1,169
American Cancer Society	Annual 45–54, Biennial 55–74	17,984	151	7.7	16.5	1,666
American College of Radiology	Annual 40–74	31,083	192	9.6	21.5	2,910

Results averaged from Models E and GE and weighted to the female population using prevalence based on the 313 SNP polygenic risk score and breast cancer family history combined. *Source including individual model results*: van den Broek et al., *J Natl Cancer Inst* 2020 [28].

^1^Age 74 was used as the age of the last screen for comparability across screening strategies for all analyses.

^2^The life years gained and breast cancer deaths averted are relative to the life years and breast cancer deaths of women at the same level of age-specific breast cancer risk who are never screened.

SNP, single nucleotide polymorphism; USPSTF, US Preventive Services Task Force.

In another example of person and health system level interactions, we used two of the CISNET models (Models E and WH) to evaluate the benefits and harms of mammography screening for women with Down syndrome, who have unique personal characteristics related to breast cancer outcomes [13]. Women with Down syndrome have lower breast cancer risk (approximately 75% lower risk of breast cancer compared to average-risk women) and significantly lower life expectancy (median 57.5 years in the US) than women without Down syndrome. In addition to estimating harms and benefits of mammography screening associated with various screening policies, we also compared the harm/benefit ratios as represented by the number of mammograms, false positives, and benign biopsies per each averted breast cancer death and life year saved. We found that the harm/benefit ratios for all evaluated mammography screening policies were consistently less favorable for women with Down syndrome than those observed for average-risk women. We found that the best harm/benefit ratios for women with Down syndrome were obtained if these women undergo one-time screening at age 50 (**Table 4**). Person-level differences in terms of risk of breast cancer and life expectancy between women with and without Down syndrome implied that health system level policies for starting and ending ages of mammography screening may need to be set differently for these women.

**Table 4:** Incremental harm/benefit ratios of various screening strategies (according to screening frequency and age) compared to no screening for average-risk women and women with Down syndrome.

Harm/benefit ratios	Average-risk women (range across models)				Women with Down syndrome (range across models)
Screening strategy	Biennial 50–74	Annual 50–74	Biennial 40–74	Annual 40–49, Biennial 50–74	Biennial 50–74
Number of mammograms per averted breast cancer death	2,240 (1,608–2,871)	5,974 (5,056–6,893)	5,412 (4,473–6,350)	7,446 (6,164–8,728)	16,735 (15,020–18,449)
Number of mammograms per life year gained	122 (99–146)	308 (278–339)	173 (149–197)	234 (204–263)	2,752 (1,670–3,835)
Number of false positives per averted breast cancer death	190 (120–260)	459 (381–537)	676 (518–833)	890 (712–1,068)	1,493 (1,406–1,580)
Number of false positives per life year gained	10 (7–13)	24 (21–26)	22 (17–26)	28 (24–32)	242 (156–328)
Number of benign biopsies per averted breast cancer death	27 (17–36)	81 (53–108)	88 (67–108)	116 (93–139)	209 (197–221)
Number of benign biopsies per life year gained	1.4 (1.0–1.8)	3.3 (2.9–3.7)	2.8 (2.2–3.4)	3.6 (3.1–4.2)	34.0 (21.9–46.0)

Results averaged from Models E and WH. *Source*: Alagoz et al., *J Gen Intern Med* 2019 [13].

Similarly, we used 2 CISNET models (Models GE and WH) to evaluate the clinical benefits and harms associated with screening guidelines for a group with unique personal characteristics [15]. Survivors of childhood cancer face high risks for early mortality and treatment-related late effects, including subsequent breast cancer [49–51]. Approximately 30% of female survivors previously treated with chest radiation will develop breast cancer before age 50, a risk similar to that of *BRCA1* mutation carriers [52]. These women also face higher competing mortality due to treatment-related late effects and a high burden of chronic diseases at an early age. [53] This competing mortality may reduce benefits from screening and treatment. Although the Children’s Oncology Group recommends early screening for breast cancer with mammography and breast MRI for these survivors starting at age 25 [54], adherence rates are low, in part due to uncertain benefits and harms.

**Fig 2:**
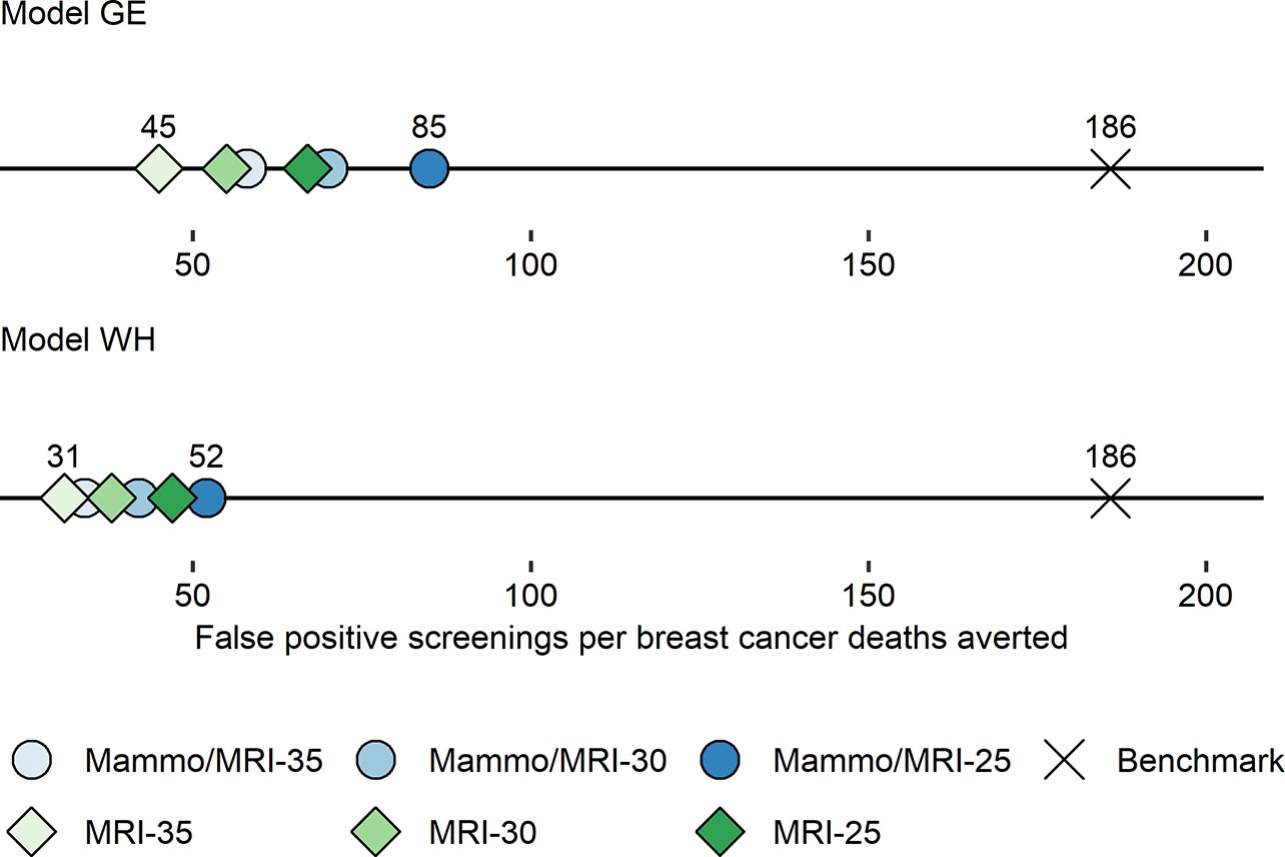
Harm/benefit ratios for breast cancer screening strategies by screening modality and starting age among childhood cancer survivors. Shown are estimates for the number of false-positive mammogram results per breast cancer death averted for each screening strategy. For context, the published benchmark estimates for the harm/benefit ratio is shown for average-risk women in the general population undergoing screening based on USPSTF recommendations (biennial mammography between 50 and 74 years of age). A lower ratio indicates a more favorable balance of harms to benefits. GE, Georgetown University Medical Center and Albert Einstein College of Medicine; Mammo, mammography; MRI, magnetic resonance imaging; USPSTF, US Preventive Services Task Force; WH, University of Wisconsin–Madison and Harvard Pilgrim Healthcare Institute. *Image source*: Yeh et al., *Ann Intern Med* 2020 [15].

To estimate the clinical outcomes associated with screening among these women, we adapted the models using data from the Childhood Cancer Survivor Study [55] to reflect the personal and tumor-level factors for the clinical course of breast cancer among these high-risk women. We estimated that among female survivors previously treated with chest radiation, nearly 60% will develop breast cancer in their lifetime. We found that early initiation of breast cancer screening could avert half or more of the expected breast cancer deaths. Similar to the work on women with Down syndrome, we compared estimated harm/benefit ratios to benchmarks for average-risk US women undergoing the US Preventive Services Task Force (USPSTF) recommendation of biennial screening between ages 50 and 74 [15]. Although the absolute number of screening tests, false positives, and benign biopsies were higher among survivors, because of the greater survival benefits, harm/benefit tradeoffs for survivors were more favorable than these benchmarks, suggesting existing guidelines are reasonable (**Fig 2**). Importantly, we found that MRI accounted for the majority of the screening benefit. Our findings highlight the importance of reducing barriers to MRI screening for these high-risk women and underscore the need to identify other options for breast cancer prevention as some survivors may prefer mammography and/or seek to reduce their risk even further. In future modeling research, we will be extending this work to evaluate the impact of healthcare system level factors such as physician recommendations for primary prevention with hormonal therapy on screening strategies and outcomes.

### Tumor, person, and health system level interactions

**Fig 3:**
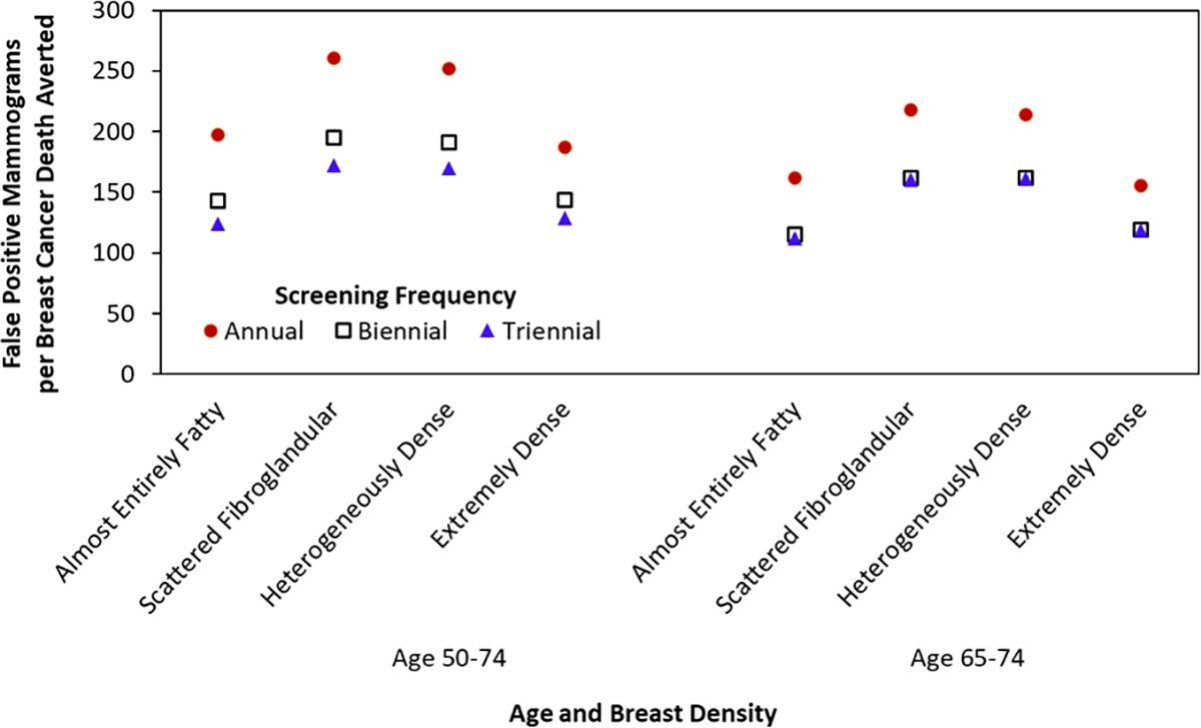
False-positive mammograms per breast cancer death averted according to age, breast density, and screening frequency among women with average breast cancer risk. Results shown from Model E compared with the scenario of no mammography screening. Values for women aged 65–74 years assume all women received biennial screening during ages 50–64 years. *Image source*: Trentham-Dietz et al., *Ann Intern Med* 2016 [9].

Modeling of 2 factors—age and breast density—are an example of modeling that incorporates interactions across all 3 levels of factors that affect breast cancer outcomes. Breast density in the CISNET models is assigned to each woman at the time she begins receiving mammography screening. As described above, density is assigned to women in 4 values (reflecting lower to higher density) and 3 age groups (**Table 1**). In certain focused analyses, risk of breast cancer incidence, and thus tumor inception for models with natural history components, depends on breast density. In these analyses, sensitivity and specificity of screening mammography also vary by breast density in addition to age, screening round (first versus subsequent), and screening frequency. For example, in an analysis parallel with modeling conducted for the USPSTF [56], 3 models (Models E, GE, and WH) compared a metric of the balance of benefits and harms from mammography screening (the ratio of false-positive mammograms to breast cancer deaths averted) according to breast density and screening frequency for 2 age groups: 50 to 64 and 65 to 74 (**Fig 3**) [9].

Findings demonstrated how, for average-risk women, moving from screening mammography every 3 years to every 2 years increases the harm/benefit ratio—as measured by false-positive mammograms per death averted—less than moving from biennial to annual screening. This harm/benefit ratio is higher for women with scattered fibroglandular and heterogeneously dense breasts than for women with almost entirely fatty or extremely dense breasts. For older women, this harm/benefit ratio is essentially equivalent for biennial and triennial screening. As screening performance improves with technological advances, such as digital breast tomosynthesis [57], the CISNET models will continue to compare the effectiveness of alternate screening strategies and their impact on the balance of benefits and harms while also accounting for the interaction between these health system developments with tumor- and person-level factors.

## Challenges and opportunities

The CISNET Breast Working Group has used multilevel systems modeling over the past 20 years to provide evidence to guide high priority public health, clinical, and individual decisions to reduce the burden of cancer. Models incorporate key features of tumors to address questions related to the biology of breast cancer and its relation to the different approaches for early detection. Person-level characteristics such as age, family history of breast cancer, polygenic risk, and breast density have also been evaluated to elucidate screening strategies that improve the balance of benefits, harms, and costs of screening. As an extension of our work focused on women of average risk, we have also examined screening and treatment approaches for special populations including survivors of childhood cancer and women with Down syndrome.

The models include the interactions between breast cancer epidemiology trends, evolution in screening technology, advances in breast cancer therapy, and new insights into the heterogeneous biology of each molecular subtype of breast cancer. The models incorporate various data types, structures and sources to reflect the tumor, person, and health system levels of data needed to portray the complexity of this common disease. As the CISNET Consortium continues its efforts to interrogate the pressing issues related to breast cancer incidence, detection, treatment and survival, additional challenges and opportunities remain to increase the impact of the consortium.

### Challenges and opportunities: Racial disparities

Racial disparities in breast cancer outcomes have persisted for decades. These disparities are substantial, including the 40% greater breast cancer mortality experienced by Black compared with White women [58]. Simulation modeling provides a unique opportunity to not only characterize disparities, but also to inform solutions. The first step in addressing racial disparities is to identify major factors across levels that explain the mortality differences between racial groups. Although many studies describe potential mediators of racial disparities in breast cancer outcomes, they often focus on individual mediators and limited subsets of the population [59]. The narrow scope and lack of generalizability often preclude such studies from explaining disparities at the population level. Simulation modeling is a valuable tool for quantifying the impact of mediators on population level disparities and to capture tumor, individual, and healthcare system level influences on outcomes. For example, CISNET studies have been used to investigate how breast cancer disparities are impacted by comorbidities. A previous CISNET study investigated the role of obesity in breast cancer disparities [10]. The study findings suggested that obesity has no net effect on Black–White breast cancer mortality disparities due to the opposing effects of obesity on pre- and postmenopausal breast cancer development. Future studies will investigate the role of key comorbidities, including diabetes, on breast cancer disparities.

CISNET models have been used to quantify the impact of breast cancer natural history, screening, and adjuvant treatment on mortality disparities [12]. This work showed that a large proportion of the Black–White mortality disparity was attributable to differences in natural history. However, this study also demonstrated that almost half of the disparity remained unexplained, suggesting that more refined modeling techniques and/or more detailed data on mediators that capture the impact of structural racism on breast cancer control are needed to explain disparities.

Recent CISNET work has begun to develop updated race-specific subtype-specific parameters and incorporate modern data on treatment disparities, enhancing the ability of CISNET models to account for present-day disparities. Future refinement of CISNET models will permit more precise attribution of mediating factors, including disparities in stage at diagnosis, screening receipt, and treatment receipt and timeliness. Enhanced modeling of mediators will, in turn, help identify the highest yield solutions for reducing disparities. Indeed, recent model updates have permitted identification of equitable screening strategies for Black women. The results of a recent CISNET study using updated model inputs demonstrated that screening may need to be initiated for Black women 5 to 10 years earlier than for White women (age 40 or 45 versus 50) to derive similar health benefits [30]. This study was made possible through the use of updated race-specific model parameters, including subtype distribution, stage at diagnosis, non-breast cancer mortality, and treatment dissemination. Future studies will be able to quantify the impact of interventions to increase access to screening, reduce diagnostic delays, and mitigate disparities in treatment.

### Challenges and opportunities: Primary prevention

Primary breast cancer prevention interventions have been underutilized [60–62], and there is limited current evaluation of their potential impact on population-level breast cancer outcomes. In our future planned work, CISNET models will be expanded to evaluate the impact of breast cancer primary prevention at the tumor, person, and health system levels. This future work builds on past CISNET research evaluating the population impact of using individual-level risk factors to tailor screening strategies such as age, high mammographic density [57,63], polygenic risk [28], exposure to therapeutic chest radiation [15], and obesity [64].

Screening is designed to detect breast cancers earlier than would have occurred in the absence of screening so that treatment confers a benefit when applied sooner, whereas breast cancer prevention strategies can be used to avoid disease and its therapies. While lifestyle changes such as avoiding obesity or reducing alcohol intake could potentially reduce risk of developing breast cancer [65–69], hormonal risk-reducing drugs like tamoxifen can prevent half of ER-positive breast cancer among women with high 5-year risk of breast cancer [70]. None of the other known prevention interventions have this magnitude of effect on avoiding breast cancer. Despite this, use of hormonal risk-reducing drugs has been low [71]. The 2019 USPSTF guidelines reiterated recommendations for clinical discussions about use of risk-reducing medication with women having a 3% or greater 5-year risk of developing breast cancer since the benefits are large and evidence of effectiveness is strong [72]. This healthcare policy-level guidance may increase physician recommendations for use of hormonal medications to prevent development of breast cancer in high-risk women. However, successful interventions to improve uptake of risk-reducing drugs will likely require policy-level data on US breast cancer trends and variation of its impact based on breast density, age, risk, and dissemination patterns at the nation level. CISNET models are well suited to synthesize data across multiple levels from high-quality data sources to provide estimates at both the population and individual level on benefits (e.g., avoiding breast cancer with risk-reducing drugs; early detection with screening) and harms (e.g., side effects; false positives) for various risk-reducing medication and screening strategies based on individual risk factors such as age and breast density.

As noted earlier, the CISNET models can also be useful to assess the potential impact of hormonal therapy for primary prevention in women who have survived childhood cancer and are at high risk for developing a subsequent breast cancer. This includes female survivors who received chest radiation [52] and potentially those who were exposed to high doses of anthracycline chemotherapy [73–77]. Risk-reducing medications could allow some survivors to avoid breast cancer entirely (versus avoiding breast cancer death via early detection with screening and treatment), but are not currently standard of care. As the rarity of childhood cancer and the long latency needed to capture subsequent cancers limit the feasibility of prospective prevention trials in survivors, model-based estimates can inform and broaden the focus of survivorship care to include primary prevention along with early detection of breast cancer. This work paves the way to expand CISNET multilevel modeling of primary prevention to include the impact of other individual-level risk factors such as alcohol intake, obesity, and physical inactivity on breast cancer outcomes.

### Challenges and opportunities: Team science and systems modeling

The CISNET Consortium embodies the key features of team science. Team science is an emerging interdisciplinary field that empirically examines scientific collaboration described as the processes by which members of research teams organize, communicate, and conduct research in an interdependent fashion [78]. Transdisciplinary team science initiatives such as CISNET have been shown to exceed long-term bibliometric indicators of scientific productivity and impact, (e.g., publication counts and journal impact factors) as compared with traditional investigator-initiated grants [79].

Some challenges typically inherent in team science have been effectively managed in CISNET, including high disciplinary diversity of membership, knowledge integration, relatively large size (40 to 50 individuals), goal alignment, geographic dispersion, and high task interdependence. Factors contributing to effective management of common challenges have included strong commitment by team members to the mission of the consortium, collegial respect, and consistent support by champions at grant funding agencies. Furthermore, reports including multiple disease models demonstrate how scientific impact is increased by transdisciplinary collaborations that reach across single organ and disease types [16,80,81].

However, other areas present opportunities for greater emphasis in future endeavors. First, “permeable boundaries” [78] across modeling teams and priorities over time force principal investigators to make difficult decisions on which projects to pursue and which to postpone or decline. Time and resources for modeling team members are consistently greater challenges to participation in a project rather than technological limitations. Modelers are frequently forced to make difficult decisions between routinely updating parameter input data (that may require time-intensive recalibration), refining and documenting program code, and pursuing answers to new scientific questions. Increased emphasis on resource sharing (see next section) holds promise to expand the number of modeling teams that can leverage CISNET models and tools to address a greater variety of pressing research questions while also continuously improving the model code and inputs and, potentially, increase the transportability of model applications to new diseases.

Second, traditional academic laboratory structure, including the demands of tenure and promotion considerations, create challenges for the support of junior modelers and early career researchers. Programmers for each modeling team usually require over a year of training prior to gaining independence for modifying software code and executing modeling scenarios within the timeline constraints of multiple ongoing projects. To address these challenges, the CISNET Consortium has increasingly added training and mentoring opportunities for junior modelers to expand the pipeline and ensure the ongoing impact of CISNET after each grant cycle ends.

### Challenges and opportunities: Resource sharing

Making the CISNET breast cancer control models accessible to the broader research community poses opportunities and challenges. Due to the complexity of the models and increasing use of the models by policy makers, it is important to increase the transparency of the models. To this end, we recently published a special issue of a journal dedicated to CISNET breast cancer models in which we described all main inputs and each individual model in great detail [5]. The models are currently operated only by the Breast Working Group, which limits their application to those questions that the group has the time and interest to work on. Efforts to share modeling resources have included the availability of 2 data visualization tools for key model inputs on CISNET’s Publication Support and Modeling Resources website [26,29,82]. One web-based tool, referred to as “Mammo OUTPuT,” assists policy makers to make informed decisions about the optimal ages of breast cancer screening initiation and is available on CISNET’s Decision Tools website [83,84]. Another decision support tool for management of cancer risk for women with BRCA mutations is included on CISNET’s Decision Tools website [83,85]. We are presently developing additional tools to guide screening and treatment decision-making. Finally, the group routinely shares model input data in publications (e.g., [86]) and has shared model code [87].

While past collaborations with policy-making bodies such as the CDC or the USPSTF have been fruitful [56,88,89], other important policy questions remain unaddressed. The ability to release the models, their most standard data inputs, and unprocessed model output to other researchers to use could greatly expand their reach and impact. However, the models were not designed with use by others in mind, and making this possible will require CISNET modelers to develop user interfaces, streamlined workflows, and documentation of how program inputs are acquired or prepared that can be understood by non-programmers. Obstacles to be overcome include computational resource intensity and restrictions on sharing of inputs that were obtained subject to data use agreements. Furthermore, policies will need to be developed regarding intellectual property rights and the integrity of the CISNET “brand.” Support for resource sharing would facilitate enhanced reach of CISNET models. Moving forward in the presence of these challenges, CISNET aims to implement open science strategies [90] to enhance model accessibility.

## Summary

In conclusion, over 2 decades, the 6 modeling teams in the CISNET Breast Working Group and their consultants and collaborators have produced numerous examples of impactful systems modeling. Ongoing work is highly likely to provide new insight into avenues for reducing the burden of breast cancer. Collateral benefits of the CISNET Consortium have included advances in modeling and simulation techniques, support of the careers of junior scientists, and the provision of research tools for the broader scientific community. CISNET researchers are committed to increasing their focus on leveraging their powerful modeling resources to reduce the long-standing racial disparities in breast cancer outcomes, and we encourage other scientists to draw on team science approaches to also use modeling for this purpose.

## References

[1] Cornelis MC, Hu FB. Systems Epidemiology: A New Direction in Nutrition and Metabolic Disease Research. Curr Nutr Rep. 2013;2(4). doi: 10.1007/s13668-013-0052-4 24278790 PMC3837346

[2] Hammond RA. Complex systems modeling for obesity research. Prev Chronic Dis. 2009;6(3):A97. 19527598 PMC2722404

[3] Dammann O, Gray P, Gressens P, Wolkenhauer O, Leviton A. Systems Epidemiology: What’s in a Name? Online J Public Health Inform. 2014;6(3):e198. doi: 10.5210/ojphi.v6i3.5571 25598870 PMC4292535

[4] Cancer Intervention and Surveillance Modeling Network, National Cancer Institute. Modeling to guide public health research and priorities. Available from: https://cisnet.cancer.gov/ Accessed August 10, 2020

[5] Alagoz O, Berry DA, de Koning HJ, Feuer EJ, Lee SJ, Plevritis SK, et al. Introduction to the Cancer Intervention and Surveillance Modeling Network (CISNET) breast cancer models. Med Decis Mak. 2018;38(1_suppl):3S–8S. doi: 10.1177/0272989X17737507 29554472 PMC5862043

[6] Cancer Intervention and Surveillance Modeling Network (CISNET) Breast Cancer Collaborators. Executive Summary. JNCI Monographs. 2006;2006(36):1–2.

[7] Berry DA, Cronin KA, Plevritis SK, Fryback DG, Clarke L, Zelen M, et al. Effect of screening and adjuvant therapy on mortality from breast cancer. N Engl J Med. 2005;353(17):1784–92. doi: 10.1056/NEJMoa050518 16251534

[8] Plevritis SK, Munoz D, Kurian AW, Stout NK, Alagoz O, Near AM, et al. Association of Screening and Treatment With Breast Cancer Mortality by Molecular Subtype in US Women, 2000–2012. JAMA. 2018;319(2):154–64. doi: 10.1001/jama.2017.19130 29318276 PMC5833658

[9] Trentham-Dietz A, Kerlikowske K, Stout NK, Miglioretti DL, Schechter CB, Ergun MA, et al. Tailoring Breast Cancer Screening Intervals by Breast Density and Risk for Women Aged 50 Years or Older: Collaborative Modeling of Screening Outcomes. Ann Intern Med. 2016;165(10):700–12. doi: 10.7326/M16-0476 27548583 PMC5125086

[10] Chang Y, Schechter CB, van Ravesteyn NT, Near AM, Heijnsdijk EA, Adams-Campbell L, et al. Collaborative modeling of the impact of obesity on race-specific breast cancer incidence and mortality. Breast Cancer Res Treat. 2012;136(3):823–35. doi: 10.1007/s10549-012-2274-3 23104221 PMC3511695

[11] Batina NG, Trentham-Dietz A, Gangnon RE, Sprague BL, Rosenberg MA, Stout NK, et al. Variation in tumor natural history contributes to racial disparities in breast cancer stage at diagnosis. Breast Cancer Res Treat. 2013;138(2):519–28. doi: 10.1007/s10549-013-2435-z 23417335 PMC3610865

[12] van Ravesteyn NT, Schechter CB. Near AM, Heijnsdijk EA, Stoto MA, Draisma G, et al. Race-specific impact of natural history, mammography screening, and adjuvant treatment on breast cancer mortality rates in the United States. Cancer Epidemiol Biomark Prev. 2011;20(1):112–22. 10.1158/1055-9965.EPI-10-0944 PMC3075821 21119071

[13] Alagoz O, Hajjar A, Chootipongchaivat S, van Ravesteyn NT, Yeh JM, Ergun MA, et al. Benefits and Harms of Mammography Screening for Women With Down Syndrome: a Collaborative Modeling Study. J Gen Intern Med. 2019. doi: 10.1007/s11606-019-05182-5 31385214 PMC6848489

[14] Stout NK, Lee SJ, Schechter CB, Kerlikowske K, Alagoz O, Berry D, et al. Benefits, harms, and costs for breast cancer screening after US implementation of digital mammography. J Natl Cancer Inst. 2014;106(6):dju092. doi: 10.1093/jnci/dju092 24872543 PMC4067109

[15] Yeh JM, Lowry KP, Schechter CB, Diller LR, Alagoz O, Armstrong GT, et al. Clinical benefits, harms and cost-effectiveness of breast cancer screening for survivors of childhood cancer treated with chest radiation: A comparative modeling study. Ann Intern Med. 2020;173(5):331–41. doi: 10.7326/M19-3481 32628531 PMC7510774

[16] Lansdorp-Vogelaar I, Gulati R, Mariotto AB, Schechter CB, de Carvalho TM, Knudsen AB, et al. Personalizing age of cancer screening cessation based on comorbid conditions: model estimates of harms and benefits. Ann Intern Med. 2014;161(2):104–12. doi: 10.7326/M13-2867 25023249 PMC4160041

[17] Lee SJ, Li X, Huang H, Zelen M. The Dana-Farber CISNET Model for Breast Cancer Screening Strategies: An Update. Med Decis Mak. 2018;38(1_suppl):44s–53s. doi: 10.1177/0272989X17741634 29554465 PMC5929104

[18] van den Broek JJ, van Ravesteyn NT, Heijnsdijk EA, de Koning HJ. Simulating the Impact of Risk-Based Screening and Treatment on Breast Cancer Outcomes with MISCAN-Fadia. Med Decis Mak. 2018;38(1_suppl):54s–65s. doi: 10.1177/0272989X17711928 29554469 PMC5862065

[19] Huang X, Li Y, Song J, Berry DA. Bayesian Simulation Model for Breast Cancer Screening, Incidence, Treatment, and Mortality. Med Decis Mak. 2018;38(1_suppl):78s–88s. 10.1177/0272989X17714473 PMC5711634 28627297

[20] Munoz DF, Xu C, Plevritis SK. Molecular Subtype-Specific Stochastic Simulation Model of US Breast Cancer Incidence, Survival, and Mortality Trends from 1975 to 2010. Med Decis Mak. 2018;38(1_suppl):89s–98s. 10.1177/0272989X17737508 PMC6538507 29554473

[21] Alagoz O, Ergun MA, Cevik M, Sprague BL, Fryback DG, Gangnon RE, et al. The University of Wisconsin Breast Cancer Epidemiology Simulation Model: An Update. Med Decis Mak. 2018;38(1_suppl):99s–111s. doi: 10.1177/0272989X17711927 29554470 PMC5862066

[22] Munoz DF, Plevritis SK. Estimating Breast Cancer Survival by Molecular Subtype in the Absence of Screening and Adjuvant Treatment. Med Decis Mak. 2018;38(1_suppl):32s–43s. doi: 10.1177/0272989X17743236 29554464 PMC6635303

[23] Surveillance, Epidemiology, and End Results (SEER) Program (www.seer.cancer.gov),. SEER*Stat Database: Incidence—SEER 9 Regs Research Data, Nov 2014 Sub (1973–2012) <Katrina/Rita Population Adjustment>—Linked to County Attributes—Total U.S., 1969–2013 Counties. Released April 2003, based on the November 2002 submission. Available from: www.seer.cancer.gov/seerstat.

[24] Mandelblatt JS, Near AM, Miglioretti DL, Munoz D, Sprague BL, Trentham-Dietz A, et al. Common Model Inputs Used in CISNET Collaborative Breast Cancer Modeling. Med Decis Mak. 2018;38(1_suppl):9S–23S. doi: 10.1177/0272989X17700624 29554466 PMC5862072

[25] CISNET. Cancer Intervention and Surveillance Modeling Network. Breast Cancer Model Profiles. Available from the National Cancer Institute at http://cisnet.cancer.gov/. Last accessed: May 2010.

[26] Gangnon RE, Sprague BL, Stout NK, Alagoz O, Weedon-Fekjaer H, Holford TR, et al. The contribution of mammography screening to breast cancer incidence trends in the United States: an updated age-period-cohort model. Cancer Epidemiol Biomark Prev. 2015;24(6):905–12. doi: 10.1158/1055-9965.EPI-14-1286 25787716 PMC4489135

[27] Munoz D, Near AM, van Ravesteyn NT, Lee SJ, Schechter CB, Alagoz O, et al. Effects of screening and systemic adjuvant therapy on ER-specific US breast cancer mortality. J Natl Cancer Inst. 2014;106(11). doi: 10.1093/jnci/dju289 25255803 PMC4271026

[28] van den Broek JJ, Schechter CB, van Ravesteyn NT, Janssens ACJW, Wolfson MC, Trentham-Dietz A, et al. Personalizing Breast Cancer Screening Based on Polygenic Risk and Family History. J Natl Cancer Inst. 2021; 113(4): 434–442. doi: 10.1093/jnci/djaa127 32853342 PMC8599807

[29] Gangnon RE, Stout NK, Alagoz O, Hampton JM, Sprague BL, Trentham-Dietz A. Contribution of Breast Cancer to Overall Mortality for US Women. Med Decis Mak. 2018;38(1_suppl):24s–31s. doi: 10.1177/0272989X17717981 29554467 PMC5963706

[30] Chapman CH, Schechter C, Trentham A, Gangnon R, Cadham C, Mandelblatt J. Would black women benefit from a screening mammography schedule that differs from that recommended for the overall population? A simulation modeling study [abstract]. In: Proceedings of the Twelfth AACR Conference on the Science of Cancer Health Disparities in Racial/Ethnic Minorities and the Medically Underserved; 2019 Sep 20-23; San Francisco, CA. Philadelphia (PA): AACR; Cancer Epidemiol Biomarkers Prev 2020;29(6 Suppl_2):Abstract nr A005.

[31] Chandler Y, Jayasekera JC, Schechter CB, Isaacs C, Cadham CJ, Mandelblatt JS. Simulation of Chemotherapy Effects in Older Breast Cancer Patients With High Recurrence Scores. J Natl Cancer Inst. 2020;112(6):574–81. doi: 10.1093/jnci/djz189 31612208 PMC7301154

[32] Early Breast Cancer Trialists’ Collaborative Group, Peto R, Davies C, Godwin J, Gray R, Pan HC, et al. Comparisons between different polychemotherapy regimens for early breast cancer: meta-analyses of long-term outcome among 100,000 women in 123 randomised trials. Lancet. 2012;379(9814):432–44. doi: 10.1016/S0140-6736(11)61625-5 22152853 PMC3273723

[33] American College of Radiology. The American College of Radiology Breast Imaging Reporting and Data System (BI-RADS). 4th ed. Reston, VA: American College of Radiology; 2003.

[34] Tice JA, Miglioretti DL, Li CS, Vachon CM, Gard CC, Kerlikowske K. Breast Density and Benign Breast Disease: Risk Assessment to Identify Women at High Risk of Breast Cancer. J Clin Oncol. 2015;33(28):3137–43. doi: 10.1200/JCO.2015.60.8869 26282663 PMC4582144

[35] Sprague BL, Gangnon RE, Burt V, Trentham-Dietz A, Hampton JM, Wellman RD, et al. Prevalence of mammographically dense breasts in the United States. J Natl Cancer Inst. 2014;106(10). doi: 10.1093/jnci/dju255 25217577 PMC4200066

[36] van den Broek JJ, van Ravesteyn NT, Mandelblatt JS, Huang H, Ergun MA, Burnside ES, et al. Comparing CISNET Breast Cancer Incidence and Mortality Predictions to Observed Clinical Trial Results of Mammography Screening from Ages 40 to 49. Med Decis Mak. 2018;38(1_suppl):140s–50s. doi: 10.1177/0272989X17718168 29554468 PMC5862071

[37] Jayasekera J, Li Y, Schechter CB, Jagsi R, Song J, White J, et al. Simulation Modeling of Cancer Clinical Trials: Application to Omitting Radiotherapy in Low-risk Breast Cancer. J Natl Cancer Inst. 2018;110(12):1360–9. doi: 10.1093/jnci/djy059 29718314 PMC6292816

[38] Jayasekera J, Schechter CB, Sparano JA, Jagsi R, White J, Chapman JW, et al. Effects of Radiotherapy in Early-Stage, Low-Recurrence Risk, Hormone- Sensitive Breast Cancer. J Natl Cancer Inst. 2018;110(12):1370–9. doi: 10.1093/jnci/djy128 30239794 PMC6292790

[39] Chandler Y, Schechter CB, Jayasekera J. Near A, O’Neill SC, Isaacs C, et al. Cost Effectiveness of Gene Expression Profile Testing in Community Practice. J Clin Oncol. 2018;36(6):554–62. doi: 10.1200/JCO.2017.74.5034 29309250 PMC5815401

[40] Oseni TO, Zhang B, Coopey SB, Gadd MA, Hughes KS, Chang DC. Twenty-Five Year Trends in the Incidence of Ductal Carcinoma in Situ in US Women. J Am Coll Surg. 2019;228(6):932–9. doi: 10.1016/j.jamcollsurg.2019.01.018 30772444

[41] Elshof LE, Tryfonidis K, Slaets L, van Leeuwen-Stok AE, Skinner VP, Dif N, et al. Feasibility of a prospective, randomised, open-label, international multicentre, phase III, non-inferiority trial to assess the safety of active surveillance for low risk ductal carcinoma in situ—The LORD study. Eur J Cancer. 2015;51(12):1497–510. doi: 10.1016/j.ejca.2015.05.008 26025767

[42] Francis A, Thomas J, Fallowfield L, Wallis M, Bartlett JM, Brookes C, et al. Addressing overtreatment of screen detected DCIS; the LORIS trial. Eur J Cancer. 2015;51(16):2296–303. doi: 10.1016/j.ejca.2015.07.017 26296293

[43] Hwang ES, Hyslop T, Lynch T, Frank E, Pinto D, Basila D, et al. The COMET (Comparison of Operative versus Monitoring and Endocrine Therapy) trial: a phase III randomised controlled clinical trial for low-risk ductal carcinoma in situ (DCIS. BMJ Open. 2019;9(3):e026797. doi: 10.1136/bmjopen-2018-026797 30862637 PMC6429899

[44] van Ravesteyn NT, van den Broek JJ, Li X, Weedon-Fekjaer H, Schechter CB, Alagoz O, et al. Modeling Ductal Carcinoma In Situ (DCIS): An Overview of CISNET Model Approaches. Med Decis Mak. 2018;38(1_suppl):126s–39s. doi: 10.1177/0272989X17729358 29554463 PMC5862063

[45] Chootipongchaivat S, van Ravesteyn NT, Li X, Huang H, Weedon-Fekjaer H, Ryser MD, et al. Modeling the natural history of ductal carcinoma in situ based on population data. Breast Cancer Res. 2020;22(1):53. doi: 10.1186/s13058-020-01287-6 32460821 PMC7251719

[46] Weedon-Fekjaer H, Li X, Lee S. Estimating the natural progression of non-invasive ductal carcinoma in situ breast cancer lesions using screening data. J Med Screen. 2020:969141320945736. doi: 10.1177/0969141320945736 32854582

[47] Lee S, Zelen M. Chapter 11: A Stochastic Model for Predicting the Mortality of Breast Cancer. J NCI Monographs. 2006;2006(36):79–86. 10.1093/jncimonographs/lgj011 17032897

[48] Mavaddat N, Michailidou K, Dennis J, Lush M, Fachal L, Lee A, et al. Polygenic Risk Scores for Prediction of Breast Cancer and Breast Cancer Subtypes. Am J Hum Genet. 2019;104(1):21–34. doi: 10.1016/j.ajhg.2018.11.002 30554720 PMC6323553

[49] Armstrong GT, Chen Y, Yasui Y, Leisenring W, Gibson TM, Mertens AC, et al. Reduction in Late Mortality among 5-Year Survivors of Childhood Cancer. N Engl J Med. 2016;374(9):833–42. doi: 10.1056/NEJMoa1510795 26761625 PMC4786452

[50] Oeffinger KC, Mertens AC, Sklar CA, Kawashima T, Hudson MM, Meadows AT, et al. Chronic health conditions in adult survivors of childhood cancer. N Engl J Med. 2006;355(15):1572–82. doi: 10.1056/NEJMsa060185 17035650

[51] Turcotte LM, Liu Q, Yasui Y, Arnold MA, Hammond S, Howell RM, et al. Temporal Trends in Treatment and Subsequent Neoplasm Risk Among 5-Year Survivors of Childhood Cancer, 1970–2015. JAMA. 2017;317(8):814–24. doi: 10.1001/jama.2017.0693 28245323 PMC5473951

[52] Moskowitz CS, Chou JF, Wolden SL, Bernstein JL, Malhotra J, Novetsky Friedman D, et al. Breast cancer after chest radiation therapy for childhood cancer. J Clin Oncol. 2014;32(21):2217–23. doi: 10.1200/JCO.2013.54.4601 24752044 PMC4100937

[53] Moskowitz CS, Chou JF, Neglia JP, Partridge AH, Howell RM, Diller LR, et al. Mortality After Breast Cancer Among Survivors of Childhood Cancer: A Report From the Childhood Cancer Survivor Study. J Clin Oncol. 2019;37(24):2120–30. doi: 10.1200/JCO.18.02219 31260644 PMC6698921

[54] Children’s Oncology Group. Long-Term Guidelines for Survivors of Childhood, Adolescent, and Young Adult Cancers. Version 5.0 (October 2018). Available from: survivorshipguidelines.org. Accessed October 1, 2020.

[55] Robison LL, Armstrong GT, Boice JD, Chow EJ, Davies SM, Donaldson SS, et al. The Childhood Cancer Survivor Study: a National Cancer Institute-supported resource for outcome and intervention research. J Clin Oncol. 2009;27(14):2308–18. doi: 10.1200/JCO.2009.22.3339 19364948 PMC2677920

[56] Mandelblatt JS, Stout NK, Schechter CB, van den Broek JJ, Miglioretti DL, Krapcho M, et al. Collaborative Modeling of the Benefits and Harms Associated With Different U.S Breast Cancer Screening Strategies. Ann Intern Med. 2016;164(4):215–25. doi: 10.7326/M15-1536 26756606 PMC5079106

[57] Lowry KP, Trentham-Dietz A, Schechter CB, Alagoz O, Barlow WE, Burnside ES, et al. Long-Term Outcomes and Cost-Effectiveness of Breast Cancer Screening With Digital Breast Tomosynthesis in the United States. J Natl Cancer Inst. 2020;112(6):582–9. doi: 10.1093/jnci/djz184 31503283 PMC7301096

[58] Tao L, Gomez SL, Keegan TH, Kurian AW, Clarke CA. Breast Cancer Mortality in African-American and Non-Hispanic White Women by Molecular Subtype and Stage at Diagnosis: A Population-Based Study. Cancer Epidemiol Biomark Prev. 2015;24(7):1039–45. doi: 10.1158/1055-9965.EPI-15-0243 25969506 PMC4490947

[59] Zavala VA, Bracci PM, Carethers JM, Carvajal-Carmona L, Coggins NB, Cruz-Correa MR, et al. Cancer health disparities in racial/ethnic minorities in the United States. Br J Cancer. 2021;124(2):315–32. doi: 10.1038/s41416-020-01038-6 32901135 PMC7852513

[60] Colditz GA, Bohlke K. Priorities for the primary prevention of breast cancer. CA Cancer J Clin. 2014;64(3):186–94. doi: 10.3322/caac.21225 24647877

[61] Freedman AN, Graubard BI, Rao SR, McCaskill-Stevens W, Ballard-Barbash R, Gail MH. Estimates of the number of US women who could benefit from tamoxifen for breast cancer chemoprevention. J Natl Cancer Inst. 2003;95(7):526–32. doi: 10.1093/jnci/95.7.526 12671020

[62] Waters EA, Cronin KA, Graubard BI, Han PK, Freedman AN. Prevalence of tamoxifen use for breast cancer chemoprevention among US women. Cancer Epidemiol Biomarkers Prev. 2010;19(2):443–6. doi: 10.1158/1055-9965.EPI-09-0930 20142242 PMC3038785

[63] Sprague BL, Stout NK, Schechter C, van Ravesteyn NT, Cevik M, Alagoz O, et al. Benefits, harms, and cost-effectiveness of supplemental ultrasonography screening for women with dense breasts. Ann Intern Med. 2015;162(3):157–66. doi: 10.7326/M14-0692 25486550 PMC4314343

[64] Mandelblatt J, van Ravesteyn N, Schechter C, Chang Y, Huang AT, Near AM, et al. Which strategies reduce breast cancer mortality most? Collaborative modeling of optimal screening, treatment, and obesity prevention. Cancer. 2013;119(14):2541–8. doi: 10.1002/cncr.28087 23625540 PMC3700651

[65] Bissell MCS, Kerlikowske K, Sprague BL, Tice JA, Gard CC, Tossas KY, et al. Breast Cancer Population Attributable Risk Proportions Associated with Body Mass Index and Breast Density by Race/Ethnicity and Menopausal Status. Cancer Epidemiol Biomark Prev. 2020;29(10):2048–56. doi: 10.1158/1055-9965.EPI-20-0358 32727722 PMC7541499

[66] Harvie M, Howell A, Evans DG. Can diet and lifestyle prevent breast cancer: what is the evidence? Am Soc Clin Oncol Educ Book. 2015:e66–73. doi: 10.14694/EdBook_AM.2015.35.e66 25993238

[67] Ligibel JA, Basen-Engquist K, Weight Management and Physical Activity for Breast Cancer Prevention and Control. Am Soc Clin Oncol Educ Book. 2019;39:e22–33. doi: 10.1200/EDBK_237423 31099634

[68] Sprague BL, Trentham-Dietz A, Egan KM, Titus-Ernstoff L, Hampton JM, Newcomb PA. Proportion of invasive breast cancer attributable to risk factors modifiable after menopause. Am J Epidemiol. 2008;168(4):404–11. doi: 10.1093/aje/kwn143 18552361 PMC2727276

[69] Tamimi RM, Spiegelman D, Smith-Warner SA, Wang M, Pazaris M, Willett WC, et al. Population Attributable Risk of Modifiable and Nonmodifiable Breast Cancer Risk Factors in Postmenopausal Breast Cancer. Am J Epidemiol. 2016;184(12):884–93. doi: 10.1093/aje/kww145 27923781 PMC5161087

[70] Fisher B, Costantino JP, Wickerham DL, Redmond CK, Kavanah M, Cronin WM, et al. Tamoxifen for prevention of breast cancer: report of the National Surgical Adjuvant Breast and Bowel Project P-1 Study. J Natl Cancer Inst. 1998;90(18):1371–88. doi: 10.1093/jnci/90.18.1371 9747868

[71] Nelson HD, Fu R, Zakher B, Pappas M, McDonagh M. Medication Use for the Risk Reduction of Primary Breast Cancer in Women: Updated Evidence Report and Systematic Review for the US Preventive Services Task Force. JAMA. 2019;322(9):868–86. doi: 10.1001/jama.2019.5780 31479143

[72] U.S. Preventive Services Task Force, Owens DK, Davidson KW, Krist AH, Barry MJ, Cabana M, et al. Medication Use to Reduce Risk of Breast Cancer: US Preventive Services Task Force Recommendation Statement. JAMA. 2019;322(9):857–67. doi: 10.1001/jama.2019.11885 31479144

[73] Ehrhardt MJ, Howell CR, Hale K, Baassiri MJ, Rodriguez C, Wilson CL, et al. Subsequent Breast Cancer in Female Childhood Cancer Survivors in the St Jude Lifetime Cohort Study (SJLIFE). J Clin Oncol. 2019;37(19):1647–56. doi: 10.1200/JCO.18.01099 31075046 PMC6804891

[74] Henderson TO, Moskowitz CS, Chou JF, Bradbury AR, Neglia JP, Dang CT, et al. Breast Cancer Risk in Childhood Cancer Survivors Without a History of Chest Radiotherapy: A Report From the Childhood Cancer Survivor Study. J Clin Oncol. 2016;34(9):910–8. doi: 10.1200/JCO.2015.62.3314 26700127 PMC4871997

[75] Teepen JC, van Leeuwen FE, Tissing WJ, van Dulmen-den Broeder E, van den Heuvel-Eibrink MM, van der Pal HJ, et al. Long-Term Risk of Subsequent Malignant Neoplasms After Treatment of Childhood Cancer in the DCOG LATER Study Cohort: Role of Chemotherapy. J Clin Oncol. 2017;35(20):2288–98. doi: 10.1200/JCO.2016.71.6902 28530852

[76] Veiga LH, Curtis RE, Morton LM, Withrow DR, Howell RM, Smith SA, et al. Association of Breast Cancer Risk After Childhood Cancer With Radiation Dose to the Breast and Anthracycline Use: A Report From the Childhood Cancer Survivor Study. JAMA Pediatr. 2019;173(12):1171–9. doi: 10.1001/jamapediatrics.2019.3807 31657853 PMC6820042

[77] Turcotte LM, Liu Q, Yasui Y, Henderson TO, Gibson TM, Leisenring W, et al. Chemotherapy and Risk of Subsequent Malignant Neoplasms in the Childhood Cancer Survivor Study Cohort. J Clin Oncol. 2019;37(34):3310–9. doi: 10.1200/JCO.19.00129 31622130 PMC7001784

[78] National Research Council. Enhancing the Effectiveness of Team Science. Washington, DC: The National Academies Press. 10.17226/19007; 2015. 26247083

[79] Hall KL, Stokols D, Stipelman BA, Vogel AL, Feng A, Masimore B, et al. Assessing the value of team science: a study comparing center- and investigator-initiated grants. Am J Prev Med. 2012;42(2):157–63. doi: 10.1016/j.amepre.2011.10.011 22261212 PMC3586819

[80] Rutter CM, Kim JJ, Meester RGS, Sprague BL, Burger EA, Zauber AG, et al. Effect of Time to Diagnostic Testing for Breast, Cervical, and Colorectal Cancer Screening Abnormalities on Screening Efficacy: A Modeling Study. Cancer Epidemiol Biomark Prev. 2018;27(2):158–64. 10.1158/1055-9965.EPI-17-0378 PMC5809257 29150480

[81] Sharpless NE. COVID-19 and cancer. Science. 2020;368(6497):1290. doi: 10.1126/science.abd3377 32554570

[82] Cancer Intervention and Surveillance Modeling Network, National Cancer Institute. CISNET Publication Support and Modeling Resources. Available from: https://resources.cisnet.cancer.gov/projects/ Accessed November 19, 2019

[83] Cancer Intervention and Surveillance Modeling Network, National Cancer Institute. CISNET Policy and Individual Decision Tools. Available from: https://cisnet.cancer.gov/resources/policy.html Accessed November 19, 2019

[84] Burnside ES, Lee SJ, Bennette C, Near AM, Alagoz O, Huang H, et al. Using Collaborative Simulation Modeling to Develop a Web-Based Tool to Support Policy-Level Decision Making About Breast Cancer Screening Initiation Age. MDM Policy Pract. 2017;2(2):Available from: doi: 10.1177/2381468317717982 29376135 PMC5785917

[85] Plevritis SK, Stanford Medicine. Decision Tool for Women with BRCA Mutations. Available from: http://brcatool.stanford.edu/ Accessed December 1, 2019

[86] Mandelblatt J, Cronin K, de Koning H, Miglioretti DL, Schechter C, Stout N. Collaborative Modeling of U.S. Breast Cancer Screening Strategies. Publication No. 14-05201-EF-4. Washington, DC: AHRQ; 2015.

[87] Vilaprinyo E, Forne C, Carles M, Sala M, Pla R, Castells X, et al. Cost-effectiveness and harm-benefit analyses of risk-based screening strategies for breast cancer. PLoS ONE. 2014;9(2):e86858. doi: 10.1371/journal.pone.0086858 24498285 PMC3911927

[88] Mandelblatt JS, Cronin KA, Bailey S, Berry DA, de Koning HJ, Draisma G, et al. Effects of mammography screening under different screening schedules: model estimates of potential benefits and harms. Ann Intern Med. 2009;151(10):738–47. doi: 10.7326/0003-4819-151-10-200911170-00010 19920274 PMC3515682

[89] van Ravesteyn NT, van Lier L, Schechter CB, Ekwueme DU, Royalty J, Miller JW, et al. Transition from film to digital mammography: impact for breast cancer screening through the National Breast and Cervical Cancer Early Detection Program. Am J Prev Med. 2015;48(5):535–42. doi: 10.1016/j.amepre.2014.11.010 25891052 PMC4405659

[90] National Academies of Sciences, Engineering, and Medicine. Open Science by Design: Realizing a Vision for 21st Century Research. Washington, DC: The National Academies Press. 10.17226/25116; 2018. 30212065

